# Inverse Relationship between Basal Pacemaker Neuron Activity and Aversive Long-Term Memory Formation in *Lymnaea stagnalis*

**DOI:** 10.3389/fncel.2016.00297

**Published:** 2017-01-04

**Authors:** Nancy Dong, Zhong-Ping Feng

**Affiliations:** Department of Physiology, University of TorontoToronto, ON, Canada

**Keywords:** *Lymnaea stagnalis*, aversive operant conditioning, spontaneously active neuron, basal neuronal activity, neuronal plasticity, individual variations in memory formation

## Abstract

Learning and memory formation are essential physiological functions. While quiescent neurons have long been the focus of investigations into the mechanisms of memory formation, there is increasing evidence that spontaneously active neurons also play key roles in this process and possess distinct rules of activity-dependent plasticity. In this study, we used a well-defined aversive learning model of aerial respiration in the mollusk *Lymnaea stagnalis* (*L. stagnalis*) to study the role of basal firing activity of the respiratory pacemaker neuron Right Pedal Dorsal 1 (RPeD1) as a determinant of aversive long-term memory (LTM) formation. We investigated the relationship between basal aerial respiration behavior and RPeD1 firing activity, and examined aversive LTM formation and neuronal plasticity in animals exhibiting different basal aerial respiration behavior. We report that animals with higher basal aerial respiration behavior exhibited early responses to operant conditioning and better aversive LTM formation. Early behavioral response to the conditioning procedure was associated with biphasic enhancements in the membrane potential, spontaneous firing activity and gain of firing response, with an early phase spanning the first 2 h after conditioning and a late phase that is observed at 24 h. Taken together, we provide the first evidence suggesting that lower neuronal activity at the time of learning may be correlated with better memory formation in spontaneously active neurons. Our findings provide new insights into the diversity of cellular rules of plasticity underlying memory formation.

## Introduction

Learning and memory are essential abilities required for the survival of organisms. The cellular and molecular mechanisms of memory formation have been largely focused on excitation-driven paradigms in quiescent neurons, which do not fire action potentials at rest. However, many brain regions relevant to learning and memory also contain spontaneously active neurons that maintain spiking activity in the absence of synaptic inputs, such as the cerebellum and basal ganglia (Häusser et al., 2004). Due to the high levels of resting activity, spontaneously active neurons possess several distinct biophysical properties, including higher intracellular Ca^2+^ levels (Muri and Knöpfel, 1994) and enhanced activation of Ca^2+^-dependent signaling molecules such as Ca^2+^/calmodulin-dependent protein kinase II (CaMKII) (Nelson et al., 2003, 2005). Accordingly, instead of excitation, synaptic inhibition is found to be a common trigger for neural plasticity in spontaneously active neurons in the cerebellum (Nelson et al., 2003, 2005; Pugh and Raman, 2008, 2009; Hull et al., 2013) and striatum (Rueda-Orozco et al., 2009) in motor learning and the ventral tegmentum area, and subthalamic nucleus in reward- and drug-related learning (Mure et al., 2012; Creed et al., 2014; Ranaldi, 2014; Weiss et al., 2014). In the cerebellar vestibular nucleus (Nelson et al., 2003, 2005) and Golgi neurons (Hull et al., 2013), reduction of tonically elevated CaMKII activity by synaptic inhibition induces persistent enhancement in firing activity. In cerebellar nuclear neurons, synaptic plasticity is triggered by the post-inhibitory rebound depolarization following synaptic inhibition from Purkinje neurons (Pugh and Raman, 2006, 2008). Taken together, these findings indicate that spontaneously active neurons likely have distinct rules of information storage and transfer that warrant further study. Elucidating the similarities and differences in the rules of plasticity between spontaneously active and quiescent neurons will expand our understanding of the full complement of activity-induced plasticity mechanisms in the nervous system.

The aversive operant conditioning of aerial respiration in the mollusk *Lymnaea stagnalis (L. stagnalis)*, the freshwater pond snail, provides an ideal model for examining inhibition-induced plasticity in spontaneously active neurons. As bimodal breathers, *L. stagnalis* exhibits aerial respiration behavior at the water surface via opening of its pulmonary orifice, the pneumostome. This breathing behavior is governed by a well-characterized central pattern generator (CPG) that consists of the interneurons Visceral Dorsal 4 (VD4), Input 3 Interneuron (IP3i) and the pacemaker neuron RPeD1, which initiates the rhythmic activity of the CPG (Syed et al., 1990; Winlow and Syed, 1992). Learned suppression of this behavior is induced by applying a tactile stimulus to the pneumostome upon each attempted opening, which activates an inhibitory synaptic input to the RPeD1 that terminates its activity and causes immediate closure of the pneumostome (Spencer et al., 2002). The resultant memory is expressed as reductions in both the total duration and frequency of pneumostome openings (Lukowiak et al., 1996). Conditioned animals exhibit short-term memory (STM; <10 min; Dalesman et al., 2013), intermediate-term memory (ITM; <3 h) and long-term memory (LTM; lasting up to 4 weeks; Lukowiak et al., 2000). The pacemaker neuron RPeD1 is the necessary site for aversive LTM formation (Scheibenstock et al., 2002), reconsolidation (Sangha et al., 2003a) and extinction (Sangha et al., 2003b). As the RPeD1 is singly identifiable and easily accessible to electrophysiological studies, the cellular mechanisms directly relevant to LTM formation can be consistently examined in each animal.

In this study, we employed the *L. stagnalis* aversive learning model to examine the relationship between basal neuronal activity and memory formation in spontaneously active neurons, and compared aversive LTM formation and learning-induced changes in action potential firing activity between animals exhibiting endogenous differences in baseline aerial respiration behavior.

## Materials and Methods

### Ethics Statement

Ethical approval is not required for research work with *L. stagnalis*.

### Animals

The great freshwater pond snails, *L. stagnalis*, were kept in aquaria at the University of Toronto, as described previously (Fei et al., 2007; Gardzinski et al., 2007). All animals used were kept in clean water on a 12 h light/12 h dark cycle at room temperature and fed lettuce three times a week. Adult snails approximately 3 months old and having a shell length of 25–30 mm were used in all experiments.

### Aversive Operant Conditioning of Aerial Respiration

**Figure 1:**
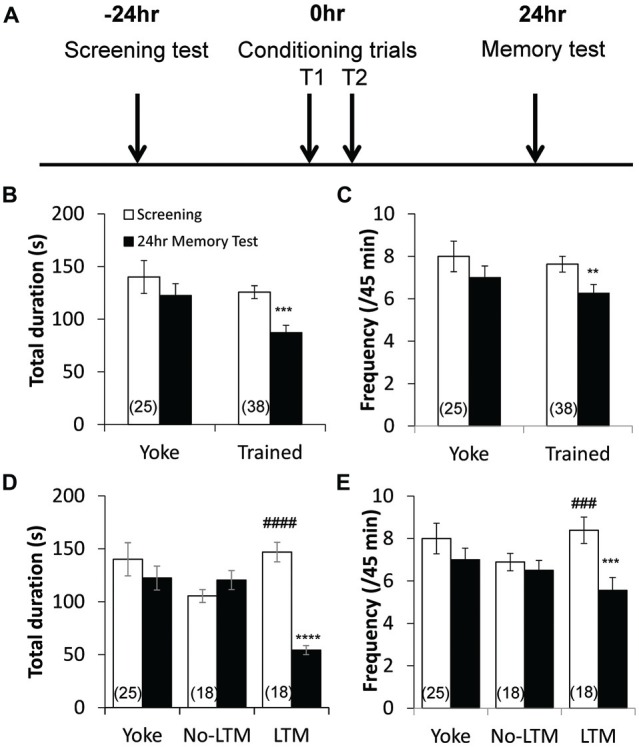
**Aversive operant conditioning results in long-term memory (LTM) formation. (A)** Time line of the conditioning paradigm. During the screening test, the frequency and total duration of pneumostome openings were observed over a 45 min period. Then, animals underwent two 45-min training sessions that were separated by an hour, during which trained animals received tactile stimuli contingent upon each attempted pneumostome opening and yoked controls received non-contingent stimuli. The frequency and total duration of pneumostome openings were observed again 24 h after conditioning during the memory test. Animals that underwent operant conditioning show significant reductions in both the total duration **(B)** and frequency **(C)** of pneumostome openings 24 h after conditioning as compared with the screening session, indicating LTM formation. No significant difference in either parameter is observed in yoked controls. In order to separate conditioned animals that do and do not form LTM, each animal was scored using the ratio of total duration of pneumostome opening at 24 h to those at screening (“D24/D0” ratio) to assess its behavioral response to the conditioning procedure. Animals that exhibit D24/D0 ratio <0.6 were classified as “LTM” and those with D24/D0 >0.8 were classified as “no-LTM”. Summary of total duration **(D)** and frequency **(E)** of pneumostome openings in yoked control, no-LTM and LTM animals before and after 24 h conditioning. Whereas LTM animals exhibit significant reductions in both the total duration and frequency of pneumostome openings at 24 h after conditioning, no-LTM animals did not. **p < 0.01, ***p < 0.001, ****p < 0.0001 as compared to screening test in LTM animals. ^###^*p* < 0.001, ^####^*p* < 0.0001 as compared to screening test in no-LTM animals.

Aversive operant conditioning protocol, modified from Guo et al. (2010), of aerial respiratory behavior under hypoxic conditions was as reported previously (Guo et al., 2010; Figure 1A). First, the frequency and total duration of hypoxia-induced pneumostome openings of each snail was measured. Eight hundred mL of dechlorinated water was placed in a 1 L beaker and bubbled with nitrogen for 20 min, resulting in hypoxic conditions (<0.1 mL O_2_/L). Individually labeled snails were placed in the hypoxic water and given 10 min to acclimate to the environment. Baseline frequency and duration of pneumostome openings were observed and recorded for 45 min. Only snails that opened their pneumostome ≥5 times were used for further experiments.

The animals were then randomly divided into two equal groups, one that underwent the operant conditioning protocol and another that served as the yoked controls. The two 45-min training sessions (T1 and T2) were conducted with an hour break in between, during which the snails were returned to their home aquaria. Aerial respiration was punished by a sharp tactile stimulus to the pneumostome each time it attempted to open (Lukowiak et al., 1996). Only animals that received ≥5 pokes in T1 were conditioned again in T2. The intensity of the stimulus was optimized such that it caused immediate closure of the pneumostome but did not elicit a complete body withdrawal response. Yoked controls received a tactile stimulus to the region surrounding the pneumostome, regardless of whether it is open, each time the snail that it was yoked with received a conditioning stimulus. The snails were returned to their home aquaria after training.

Twenty-four hours after conditioning, a 45-min memory test was performed using identical protocol as screening test. The experimenter was blind to the type of conditioning received by each animal. The snails were returned to their home aquaria after the memory test. LTM was defined as statistically significant (*P* < 0.05) reduction in both the frequency and total duration of pneumostome openings measured between the screening and memory tests (Guo et al., 2010).

### Tissue Preparations

Snails were anesthetized with 10% Listerine for 7 min and de-shelled with curved forceps. The tissue preparations were prepared in snail saline (mM: NaCl 51.3, KCl 1.7, CaCl_2_ 4.1, MgCl_2_ 1.5, HEPES 2, adjusted to pH 7.9 using NaOH). Before recording, the outer sheath surrounding the ganglia of the tissue preparations was removed using fine forceps. The preparations were then digested with a 1:5 dilution of trypsin (type III, Sigma, ON, Canada) for 4 min and rinsed 3 times with snail saline to remove the inner membranous sheath.

In order to eliminate synaptic inputs from the periphery to the RPeD1, isolated preparations of the central ring ganglia were dissected as described previously (Elliott and Benjamin, 1989; Feng et al., 2009; Nejatbakhsh et al., 2011). The central ring ganglia and buccal ganglia were isolated from the body and pinned dorsal side up on a dissecting board filled with snail saline. The entire preparation was submerged in snail saline.

### Conventional Sharp-Electrode Intracellular Recordings

Conventional sharp-electrode intracellular recording techniques were used to study the neuronal activities of RPeD1 (Syed and Winlow, 1991; Spencer et al., 1999). Specifically, sharp microelectrodes were pulled on a Flaming-Brown micropipette puller (Model P-97, Sutter Instruments, Novato, CA, USA) and filled with saturated K_2_SO_4_ solution (resistance ~20–30 MΩ). Identified RPeD1 were impaled and signals were amplified with Axopatch-1D connected to a Digidata 1200 interface controlled by pClampex 8 (Axon Instruments, Foster City, CA, USA). Data was filtered at 1 kHz and digitized at a sampling frequency of 10 kHz. All recordings were carried out in the RPeD1 in isolated preparations bathed in snail saline at room temperature (~22°C). Electrode resistance was measured at the start and end of each experiment.

Spontaneous and evoked firing activities of RPeD1 were measured in the isolated preparations of the central ganglia ring (Spencer et al., 1999). After impalement of the RPeD1, spontaneous firing activity of the neuron was measured for 10 min. The evoked activities were measured following the current step protocol, from hyperpolarizing current to depolarizing currents in successive 50 pA depolarizing steps for 60 s each, separated by 10 s breaks. A square-wave hyperpolarizing 1 nA current was injected at the beginning and end of the recordings to estimate the input resistance.

### Data Analysis

All analyses of recordings were carried out using Clampfit 9.2 (Axon Instruments). The membrane potential and spontaneous firing frequency were determined at the end of the 10-min rest period. The gain of spike firing frequencies was calculated from the slope of the linear function between the frequency of action potentials elicited at each current step and the injected current (Nelson et al., 2003). A high gain of firing is interpreted as a high neuronal firing response, and vice versa. The input resistance was calculated according to Ohm’s law from the steady state change in membrane potential in response to the injected hyperpolarizing 1 nA current.

### Statistical Analysis

The data are presented as the mean ± SEM. Statistical analyses were carried out using GraphPad Prism 6 (GraphPad Software, La Jolla, CA, USA). Differences between mean values before and after conditioning for each experimental group were tested using two-tail paired Student’s *t* test for two groups. One-way analysis of variance (ANOVA) followed by the Holm-Sidak *post hoc* test was used for multiple comparisons. Differences were considered statistically significant if *P* < 0.05.

## Results

### Operant Conditioning Results in LTM Formation

The pre-screened animals received operant or yoke conditioning, following the protocols (Figure 1A) previously described by Guo et al. (2010). The aerial respiratory behavioral activities (pneumostome openings) of the animals were then measured 24 h after the conditioning procedure. Operant conditioned animals (*n* = 38) exhibited significant reductions in both the total duration (*P* < 0.001; Figure 1B) and frequency (*P* < 0.01; Figure 1C) of pneumostome openings as compared to baseline, indicating LTM formation. In contrast, the yoked controls (*n* = 25) exhibited no significant difference in the total duration (*P* > 0.05; Figure 1B) or frequency (*P* > 0.05; Figure 1C) after conditioning as compared to baseline.

### Separation of Conditioned Animals into no-LTM and LTM Groups

In order to examine the relationship between basal RPeD1 firing activity and aversive LTM formation ability, we first sought to separate animals that, despite undergoing the same conditioning paradigm, did and did not form LTM. Each animal was scored using the ratio of total duration of pneumostome opening at 24 h to those at screening (“D24/D0” ratio) to assess its behavioral response to the conditioning procedure. Animals that exhibited D24/D0 ratio <0.6, i.e., more than 40% reduction in the total duration of pneumostome openings, were classified as “LTM”, and those with D24/D0 >0.8 were classified as “no-LTM”. Of the 38 trained animals (Figures 1B,C), 18 belonged to “LTM” group and 18 to the “no-LTM” group. The two animals with D24/D0 ratios between 0.6 and 0.8 were excluded from data analysis in this section. The total duration and frequency of pneumostome opening behavior of yoked control, no-LTM and LTM animals before and 24 h after conditioning are summarized in Figures 1D,E, respectively. Whereas animals labeled as “no-LTM” exhibited no significant changes in the total duration (*P* < 0.05) and frequency (*P* < 0.05) of pneumostome openings after conditioning, both parameters of the aerial respiration behavior were significantly reduced in animals labeled as “LTM” (total duration: *P* < 0.0001; frequency: *P* < 0.0005). Our findings indicate that there was natural variation in aversive LTM formation within a healthy and genetically homogenous adult *L. stagnalis* population.

### Only Animals with LTM Exhibit Enhanced RPeD1 Firing Activity at 24 h After Conditioning

**Figure 2:**
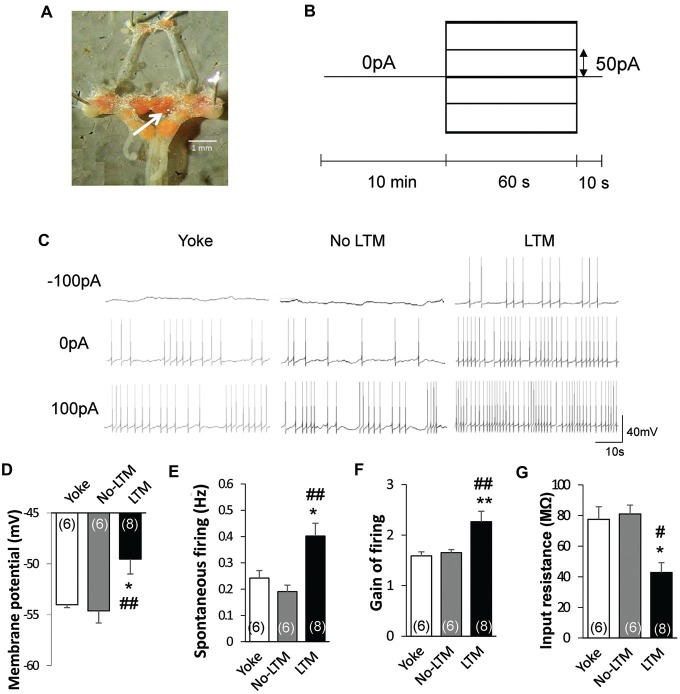
**LTM is associated with enhanced RPeD1 firing activity. (A)** Intracellular sharp electrode recordings of the RPeD1 were performed in isolated preparations of the central ganglia ring to measure its membrane potential, spontaneous firing frequency, gain of firing and input resistance as indices of neuronal firing properties. White arrow indicates the location of RPeD1. **(B)** The evoked activity step protocol. **(C)** Representative traces of RPeD1 firing upon current injection in no-LTM, LTM and yoke animals. Animals with LTM exhibit significantly depolarized membrane potential **(D)**, increased spontaneous firing frequency **(E)**, enhanced gain of firing **(F)** and reduced input resistance **(G)** as compared to no-LTM and yoked control animals, whereas the latter two groups do not differ from each other. *p < 0.05, **p < 0.01, as compared to yoked control. ^#^*p* < 0.05, ^##^*p* < 0.01 as compared to no-LTM.

To determine the relationship between learning behavior and neuronal activity, we next performed intracellular sharp electrode recordings of the RPeD1 in no-LTM and LTM animals to examine its basal membrane properties and firing activity. Isolated preparations of the central ring ganglia (Figure 2A) were extracted from randomly chosen yoked control (*n* = 6), no-LTM (*n* = 6) and LTM (*n* = 8) animals immediately after the 24 h memory test. In each preparation, spontaneous activity of the RPeD1 was first recorded for 10 min after impalement in order to measure the membrane potential and spontaneous firing frequency as indices of RPeD1 firing activity at rest (Figures 2B,C). Then an evoked activity step protocol was applied to measure the gain of RPeD1 firing response to injected current as an index of RPeD1 evoked firing activity (Nelson et al., 2003; Figures 2B,C). The resultant firing frequencies were plotted against the current injected in order to calculate the slope of the linear relationship, which was taken as the “gain of firing”, where a higher value indicated greater firing response to inputs. We found that membrane potential was more depolarized in LTM animals as compared to the no-LTM (*P* < 0.01) and yoked control animals (*P* < 0.05), with no difference between the latter two groups (*P* > 0.05; Figure 2D). Consistent with a more depolarized membrane potential, the frequency of spontaneous firing was also significantly higher in LTM animals than in no-LTM (*P* < 0.001) and yoked control animals (*P* < 0.05), with no difference between the latter two groups (*P* > 0.05; Figure 2E). The RPeD1 in animals with LTM exhibited significantly greater gain of firing than no-LTM (*P* < 0.01) and yoked control animals (*P* < 0.01), with no difference between the latter two groups (*P* > 0.05; Figure 2F). The input resistance of a neuron is an inverse measurement of the membrane conductance, such that a low input resistance reflects a high conductance implying opening of membrane ion channels. We thus compared the input resistance of the RPeD1 from the different groups. We found that the input resistance of RPeD1 in animals with LTM decreased significantly as compared to no-LTM (*P* < 0.05) and yoked control animals (*P* < 0.05), with no difference between the latter two groups (*P* > 0.05; Figure 2G). Together, these findings indicate that the RPeD1 in no-LTM and LTM animals differ in their basal membrane properties and firing activity at 24 h after conditioning.

### Naïve Animals with Lower Aerial Respiration Behavior Exhibit Higher RPeD1 Firing Activity

**Figure 3:**
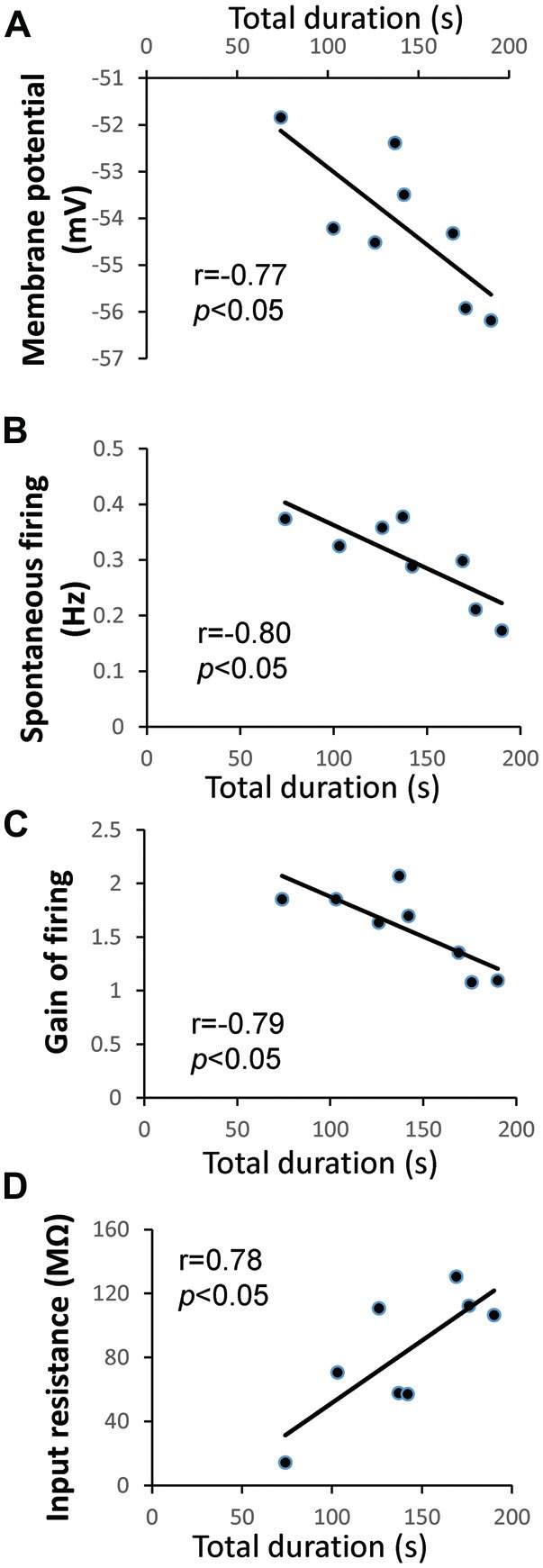
**Relationship between aerial respiration behavior and RPeD1 firing activity in naïve animals. (A)** Intracellular sharp electrode recordings of the RPeD1 were performed in isolated preparations of the central ring ganglia. Correlations between total duration of pneumostome openings and membrane potential **(A)**, spontaneous firing frequency **(B)**, gain of firing **(C)** and input resistance **(D)**.

Animals in the “LTM” group exhibited higher baseline total duration (*P* < 0.0001) and frequency (*P* < 0.001) of pneumostome openings than animals in the “no-LTM” group, even though as naïve animals they were chosen randomly for screening and conditioning (Figures 1D,E). Thus, we used the baseline aerial respiration behavior as a predictive biomarker for aversive LTM formation ability, and then examined the relationship between basal RPeD1 firing activity and aerial respiration behavior in naïve animals to gain insights into its role in determining aversive LTM formation (*n* = 8). Specifically, random naive animals underwent a 45 min screening test to measure their baseline aerial respiration behavior and then were sacrificed to extract isolated preparations of the central ring ganglia (Figure 2A) for electrophysiological recordings. The membrane potential, spontaneous firing frequency, gain of firing and input resistance of each RPeD1 were measured as described above (Figure 2B) and correlated to the total duration of pneumostome openings observed in that animal during the screening test. We found that RPeD1 membrane potential (linear regression, *P* < 0.05; Figure 3A), spontaneous firing frequency (linear regression, *P* < 0.05; Figure 3B) and gain of firing (linear regression, *P* < 0.05; Figure 3C) were lower in the animals with longer total pneumostome opening duration. In contrast, input resistance of the RPeD1 was higher in animals with longer total duration of pneumostome openings (linear regression, *P* < 0.05; Figure 3D). These findings indicate that RPeD1 firing activity was negatively correlated with aerial respiration behavior in naïve animals.

### Differences in Baseline Aerial Respiration Behavior Is Associated with Different Responses to Aversive Operant Conditioning

**Figure 4:**
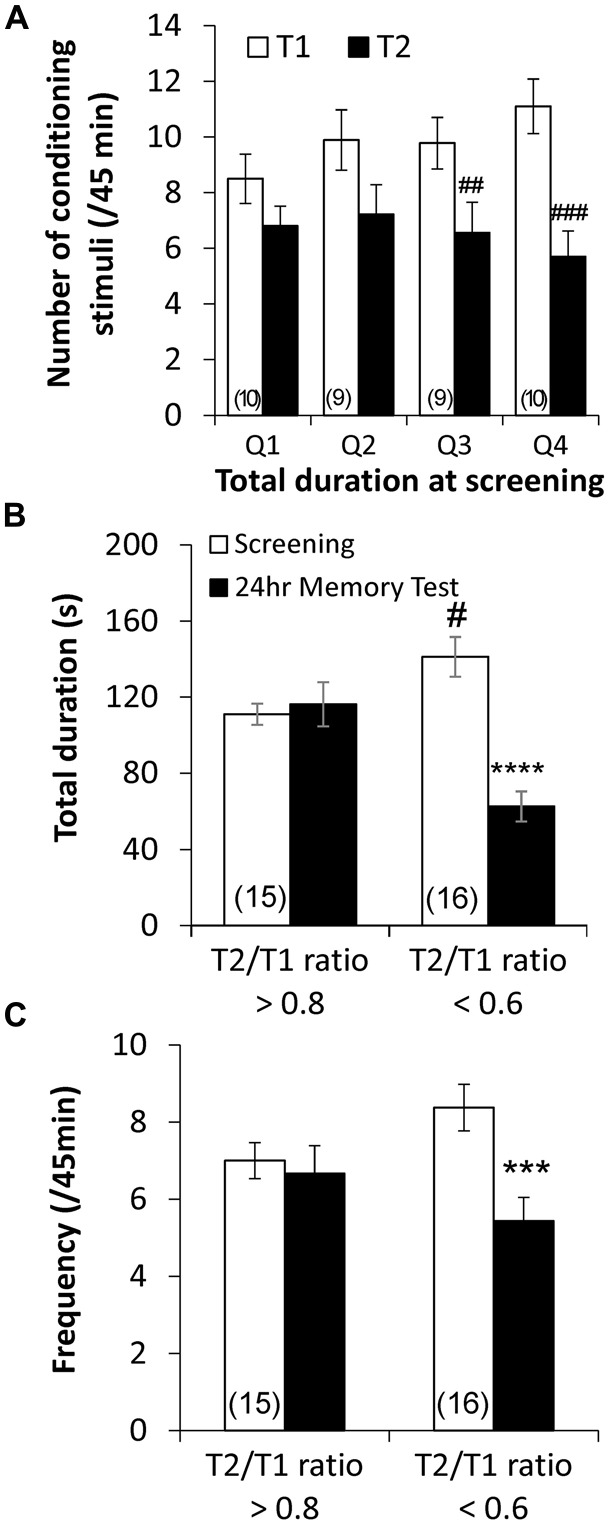
**Endogenous differences in basal aerial respiration behavior is correlated with different responses to aversive operant conditioning. (A)** Quartile analysis of behavioral data from trained animals demonstrated that animals whose basal total duration of aerial respiration behavior was in the upper two quartile exhibited significant reductions in the number of conditioning stimuli received during the second training session (T2) as compared to the first (T1), whereas animals in the lower two quartile did not. Retrospective analysis using the average ratio of number of conditioning stimuli received during T2 to those during T1 (“T2/T1 ratio”) in animals in the upper two quartiles, ~0.6, and lower two quartiles, ~0.8, as the criteria showed that animals that exhibited T2/T1 ratio <0.6 showed reduced total duration **(B)** and frequency **(C)** of pneumostome opening at 24 h after conditioning, whereas those that exhibited T2/T1 ratio >0.8 did not. ^##^*p* < 0.01, ^###^*p* < 0.001 as compared to T1. ***p < 0.001, ****p < 0.0001 as compared to screening test. ^#^*p* < 0.05 as compared to screening test level in T2/T1 ratio >0.8 animals.

To further examine the role of baseline RPeD1 firing activity as a determinant of aversive LTM formation, we next studied the relationship between baseline aerial respiration behavior, as a proxy measure of baseline RPeD1 firing activity, and response of animals to the conditioning procedure. Behavioral data of trained animals (Figure 1) were separated into quartiles according to the baseline total duration of pneumostome opening and the number of conditioning stimuli received during the two conditioning sessions (T1 and T2) were compared across the quartiles (Figure 4A). We found that animals whose baseline total duration of aerial respiration were in the upper two quartiles exhibited significant reductions in the number of conditioning stimuli received during T2 than during T1 (Q3: *n* = 9, *P* < 0.01; Q4: *n* = 10, *P* < 0.001), whereas those in the lower two quartiles did not (Q1: *n* = 10, *P* > 0.05; Q2: *n* = 9, *P* > 0.05). However, the four groups of animals received similar numbers of conditioning stimuli during T1 (*P* > 0.05).

To confirm whether animals that responded differently to the conditioning procedure indeed differed in aversive LTM formation, we regrouped behavioral data of the trained animals based on the average T2/T1 ratio of number of received conditioning stimuli observed in animals in the upper two quartiles (*n* = 19) and in animals in the lower two quartiles (*n* = 19). Re-analysis of the behavioral data (Figure 1) showed that the trained animals that exhibited a T2/T1 ratio <0.6 (*n* = 16) showed significant reductions in both the total duration (*P* < 0.0001; Figure 4B) and frequency (*P* < 0.001; Figure 4C) of pneumostome openings 24 h after conditioning, which were indicative of LTM formation (Guo et al., 2010). Trained animals that exhibited a T2/T1 ratio >0.8 (*n* = 15) did not exhibit significant differences in either the total duration (*P* > 0.05; Figure 4B) or frequency (*P* > 0.05; Figure 4C) of pneumostome openings at 24 h after conditioning. The animals that exhibited a T2/T1 ratio <0.6 still showed significantly higher baseline total duration of pneumostome openings than the animals with T2/T1 ratio >0.8 (*P* < 0.05; Figure 4B). Taken together, these findings further indicate that animals with endogenous differences in aerial respiration behavior exhibited different responses to aversive operant conditioning.

### RPeD1 Firing Activity Is Modulated in a Biphasic Manner during LTM Formation

Finally, we sought to examine the cellular consequences of the observed differences in responses to conditioning on the aversive LTM formation process. Labeling animals that exhibited T2/T1 ratio <0.6 as “LTM” and T2/T1 >0.8 as “no-LTM”, we characterizing changes in RPeD1 firing activity in these two groups of animals during the first 24 h after conditioning. After animals underwent two 45-min training sessions, isolated preparations of the central ring ganglia were extracted from trained animals at 1, 2, 3, 4–6, 6–8, 8–10 and 24 h after the end of the second training session (T2). The trained animals were grouped into no-LTM and LTM groups based on the T2/T1 criteria. Electrophysiological recordings were carried out to measure the membrane potential, spontaneous firing frequency, gain of firing and input resistance as indices of neuronal firing properties of the RPeD1.

**Figure 5:**
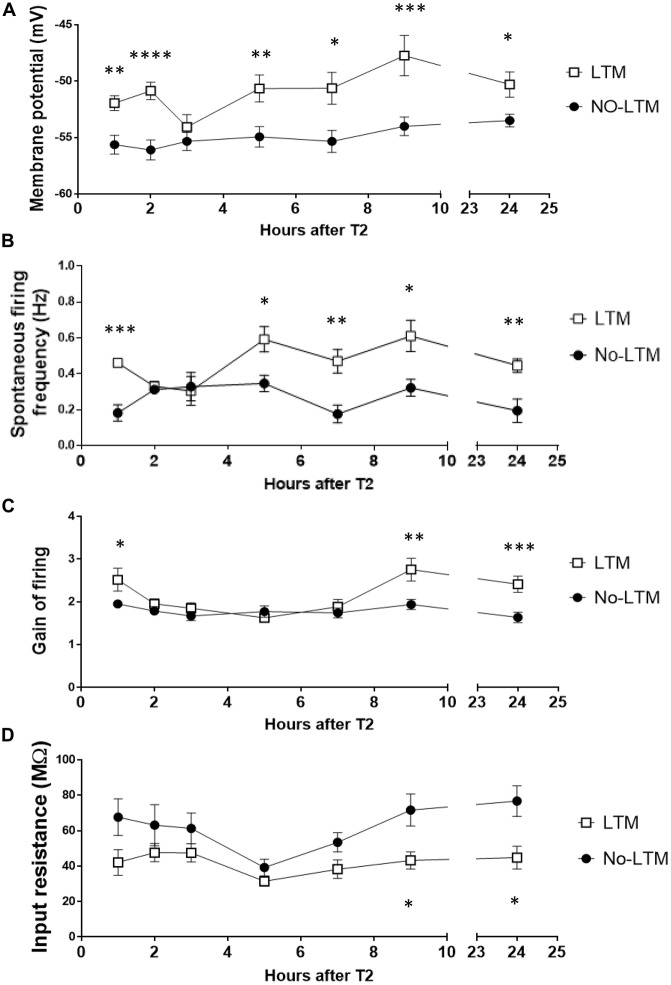
**RPeD1 firing activity is enhanced in a time-dependent manner during the first 24 h after conditioning in animals that form LTM.** Central ring ganglia of no-LTM and LTM animals were extracted at various time points during the first 24 h after conditioning. Intracellular sharp electrode recording of the RPeD1 was used to measure its membrane potential **(A)**, spontaneous firing frequency **(B)**, gain of firing **(C)** and input resistance **(D)**. As compared to no-LTM animals (1 h: *n* = 5; 2 h: *n* = 6; 3 h: *n* = 8; 4–6 h: *n* = 8; 6–8 h: *n* = 6; 8–10 h: *n* = 6; 24 h: *n* = 8), RPeD1 of animals with LTM (1 h: *n* = 6; 2 h: *n* = 6; 3 h: *n* = 6; 4–6 h: *n* = 6; 6–8 h: *n* = 6; 8–10 h: *n* = 6; 24 h: *n* = 8) exhibit two temporally discontinuous phases of enhancement in membrane potential, spontaneous firing frequency and gain of firing: an early phase that decays within 3 h and a late phase that persists by 24 h. Input resistance in LTM animals is lower than that of no-LTM animals at 8–10 and 24 h. *p < 0.05, **p < 0.01, ***p < 0.001, ****p < 0.0001.

Figure 5A shows the time-dependent changes in RPeD1 membrane potential during aversive LTM formation. LTM animals exhibited a biphasic depolarization in membrane potential as compared to no-LTM animals. The early phase of enhancement spanned the first 2 h after conditioning (1 h: *P* < 0.005; 2 h: *P* < 0.0001). Starting at 4–6 h, the membrane potential underwent a second phase of enhancement that persisted by 24 h (4–6 h: *P* < 0.01; 6–8 h: *P* < 0.05; 8–10 h: *P* < 0.001; 24 h: *P* < 0.01). Spontaneous firing activity showed similar biphasic enhancement in LTM animals relative to the no-LTM animals (Figure 5B). The first phase of enhancement in the LTM group occurred during the first hour after conditioning (*P* < 0.0005). The late phase of enhanced spontaneous firing appeared at 4–6 h and persisted at 24 h (4–6 h: *P* < 0.05; 6–8 h: *P* < 0.005; 8–10 h: *P* < 0.05; 24 h: *P* < 0.005). The gain of firing was enhanced in two temporally discontinuous phases in LTM as compared to no-LTM animals (Figure 5C). The first phase of enhancement in LTM animals occurred during the first hour after conditioning (*P* < 0.05). The second phase appeared later at 8–10 h (*P* < 0.005) and persisted at 24 h (*P* < 0.0005). The dynamics of input resistance are shown in Figure 5D. At 8–10 h (*P* < 0.05) and 24 h (*P* < 0.05), input resistance in LTM animals was significantly lower than no-LTM animals. Taken together, we provide the first description of the dynamic changes in RPeD1 firing pattern following aversive operant conditioning. These results demonstrate that the RPeD1 in no-LTM and LTM animals exhibited distinct cellular responses to the conditioning procedure during the first 24 h after aversive operant conditioning.

## Discussion

To investigate the relationship between basal neuronal activity and memory formation in spontaneously active neurons, we compared learning-induced behavioral and cellular changes in animals exhibiting endogenous differences in baseline aerial respiration behavior in an aversive operant conditioning paradigm in *L. stagnalis*. We found that aerial respiration behavior is negatively correlated with RPeD1 firing activity in naïve animals. The higher baseline aerial respiration is associated with early response to the conditioning procedure and better aversive LTM formation. These findings suggest that lower neuronal activity in spontaneously active neurons at the time of learning may facilitate memory formation.

### Individual Differences in RPeD1 Membrane Properties and Firing Activity

Recent studies have shown considerable biological variations in neuronal properties, such as basal CREB phosphorylation (Cowansage et al., 2013) and afterhyperpolization profile (Moore et al., 2011), in healthy and genetically homogenous animals. Here, we observed that individual differences in aerial respiration behavior in an inbred population of *L. stagnalis* are associated with differences in both basal and active membrane properties of the RPeD1. As the intrinsic electrophysiological properties of a neuron are largely determined by the ion channels present on the membrane (Magee, 2000), it is likely that there are endogenous differences in the expression and/or function of ion channels expressed by the RPeD1.

**Figure 6:**
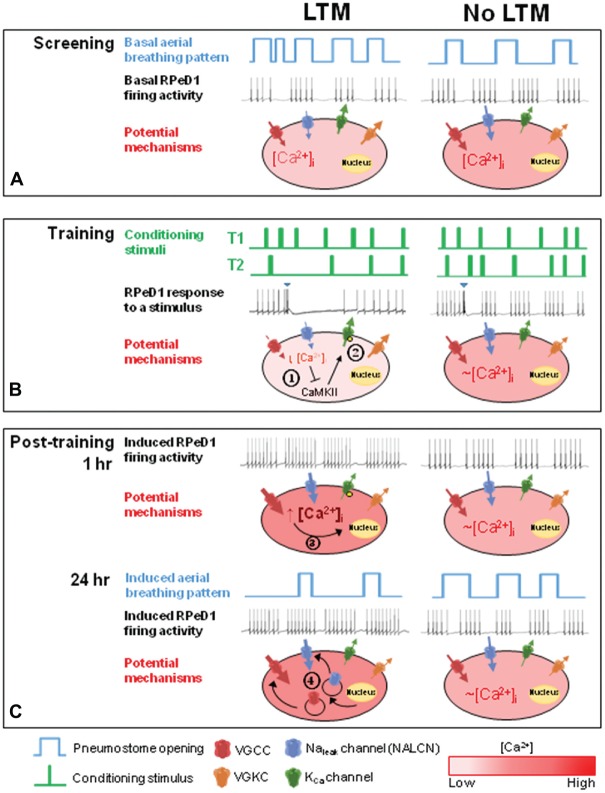
**Proposed model describing the role of basal RPeD1 firing activity in aversive LTM formation. (A)** In naïve animals, differences in endogenous expression and/or activity level of several ion channels may underlie individual variations in basal RPeD1 rhythmic firing activity, which is negatively correlated with basal aerial respiration behavior. Animals that form aversive LTM exhibit lower basal RPeD1 firing activity and higher basal aerial respiration behavior than animals that do not form aversive LTM. **(B)** During training, animals with high- and low-firing RPeD1s exhibit different responses to the conditioning stimuli, as reflected in the different number of conditioning stimuli delivered in T2. Application of a conditioning stimulus (blue inverted triangle) to the open pneumostome results in a transient inhibition of RPeD1 firing activity. The inhibitory input strongly suppresses spike firing in a RPeD1 with lower basal firing activity, resulting in a reduction in intracellular Ca^2+^ concentration that has been shown to trigger inhibition of CaMKII (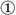) and K_Ca_ channel activity (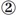) (potential modifications signified by yellow circle), and potentiation of firing activity (Nelson et al., 2003, 2005) **(C)**. In contrast, the conditioning stimulus does not significantly induce changes in the spike firing and intracellular Ca^2+^ concentration in a RPeD1 with higher basal firing activity, such that cellular changes underlying aversive LTM formation do not occur **(C)**. **(C)** In animals exhibiting low basal RPeD1 firing, the training-induced enhancement in RPeD1 rhythmic firing activity observed at 1 h after training could induce increases in intracellular Ca^2+^ concentration and consequently activity-dependent signaling pathways (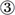), such as CREB (Guo et al., 2010), that trigger aversive LTM formation. At 24 h after training, *de novo* synthesis of plasticity products (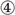), such as voltage-gated Ca^2+^ and non-selective Na^+^ leak channel (NALCN) leak channels, may serve to maintain enhanced RPeD1 firing activity and aversive LTM.

Basal membrane potential of neurons is largely determined by K^+^ and Na^+^ leak currents (Hodgkin and Huxley, 1947; Lu et al., 2007), mediated by channels including the two-pore domain K^+^ channels (Enyedi and Czirjak, 2010), the non-selective Na^+^ leak channel (NALCN; Lu et al., 2007) and the hyperpolarization-activated cyclic nucleotide–gated (HCN) channels (Kase and Imoto, 2012). In particular, the molluskan ortholog of the NALCN channel, the U-type channel, conducts the Na^+^ leak current that critically regulates RPeD1 membrane potential (Lu and Feng, 2011; Lu et al., 2016). Our finding that more depolarized RPeD1 membrane potential is associated with lower input resistance, and thus greater net inward current, suggests that differences in U-type channel-mediated Na^+^ leak current may in part underlie the observed endogenous differences in RPeD1 basal membrane properties. The individual variations in active membrane properties of the RPeD1, i.e., the spontaneous firing frequency and gain of firing, may be attributable to differences in the expression and/or function of the voltage-gated K^+^ (Johnston et al., 2010) and Ca^2+^ (Simms and Zamponi, 2014) channels and big-conductance (BK) Ca^2+^-dependent K^+^ channels (Lee and Cui, 2010; Dong and Feng, 2016) that are active during the action potential and have been implicated in controlling firing frequency (Figure 6). These possibilities are consistent with a recent study demonstrating that differential expression of Kv4.2 and HCN channels underlie differences in the electrophysiological behaviors of hippocampal CA1 pyranimdal neurons along the longitudinal hippocampal axis (Marcelin et al., 2012).

The sources of such inter-individual variations within a genetically homogenous population are currently unclear. The naïve animals used this study were randomly chosen from an inbred population that is raised under the same conditions, i.e., temperature, water quality, food source, *etc*. Interestingly, evidence from inbred mice raised in strictly controlled environments have shown that ~80% of random variability in quantitative traits, such as body weight, are unrelated to genetic and environmental influences (Gärtner, 2012). Epigenetic modifications, such as histone acetylation or methylation, have been proposed to contribute to this component of biological variations (Wong et al., 2005; Dulac, 2010).

### Neuronal Activity and Aerial Respiration Behavior

Understanding the relationship between activity of spontaneously active neurons and their behavioral output remains a central task in neuroscience research. The neuronal network of aerial respiration model in *L. stagnalis* provides an ideal model for such a purpose, as the giant respiratory pacemaker neuron RPeD1 required for initiating respiratory rhythm (Syed et al., 1990) is singly identifiable in each animal and easily accessible to electrophysiological studies. McComb et al. (2003) have previously demonstrated, in semi-intact preparations of the *L. stagnalis* central ring ganglia, that naïve juvenile animals (~7.5 weeks old) exhibit higher RPeD1 firing activity and lower aerial respiration activity as compared to older adult animals (~13 weeks). In this study using isolated preparations of the central ring ganglia extracted from adult animals (~3 months), we found a similarly inverse relationship between RPeD1 firing activity and aerial respiration behavior. This is consistent with our observations that reduced aerial respiration behavior after aversive operant conditioning is correlated with enhanced RPeD1 firing activity. Learning-induced enhancements in neuronal activity have also been shown in the conditioned taste aversion paradigm in *L. stagnalis* (Jones et al., 2003; Kemenes et al., 2006; Nikitin et al., 2008). An earlier study demonstrated that learned suppression of aerial respiration behavior was associated with reduced RPeD1 activity (Spencer et al., 1999). Further studies are needed to better understand the basis for these different observations. In this study, we employed the isolated central ring ganglia to study the intrinsic firing properties of the RPeD1 in the absence of peripheral inputs (Inoue et al., 2001). Thus, the RPeD1 exerts an overall inhibitory effect on pneumostome opening behavior, and higher RPeD1 firing activity results in greater suppression of IP3i and VD4 activity, thereby preventing pneumostome openings.

### Relationship Between Basal RPeD1 Neuronal Activity and Aversive LTM Formation

The rules of plasticity in spontaneously active neurons may differ significantly from those in quiescent neurons (Nelson et al., 2003, 2005; Pugh and Raman, 2006, 2008; Hull et al., 2013). As spontaneously active neurons play key roles in motor and reward learning (Nelson et al., 2003, 2005; Pugh and Raman, 2008, 2009; Rueda-Orozco et al., 2009; Mure et al., 2012; Hull et al., 2013; Creed et al., 2014; Ranaldi, 2014; Weiss et al., 2014), better understanding of the full complement of plasticity mechanisms in the brain can have far-reaching impacts on addressing motor disorders and addiction. This study examined the role of basal neuronal activity of spontaneously active neurons in inhibition-induced memory formation. We demonstrated that lower basal RPeD1 firing activity at the time of conditioning, as mediated by low basal net membrane conductance, may be associated with stronger responses to operant learning and better aversive LTM formation, resulting in increased RPeD1 firing and higher net membrane conductance as observed by the lower input resistance. This is consistent with a previous study comparing two strains of *L. stagnalis* with disparate aversive LTM formation abilities, showing in the semi-intact preparation of the central ring ganglia that the RPeD1 in animals from the “smarter” strain exhibit lower baseline activity and lower input resistance (Braun et al., 2012). We expand upon these findings by demonstrating that differences in basal RPeD1 firing activity may also underlie variations in learning ability within a healthy and genetically homogenous population.

In contrast, numerous studies have shown in various animal models that *higher* neuronal activity at the time of learning facilitates memory formation (Saar et al., 1998; Stackman et al., 2002; Zelcer et al., 2006; Han et al., 2009; Santini and Porter, 2010; Santini et al., 2012; Volle et al., 2016). This discrepancy may in part be because LTM formation in this paradigm is induced by *inhibitory* inputs, elicited by the conditioning stimuli, to the RPeD1. Electrophysiologically silencing RPeD1 spike firing in between training sessions reduced the number of sessions required to produce LTM (Lowe and Spencer, 2006). Studies in other models of inhibition-induced neural plasticity have also shown that effective inhibition of firing activity in the postsynaptic neuron is key to triggering the biochemical changes that result in plasticity. In an interesting parallel to the *L. stagnalis* model, brief periods of synaptic inhibition can trigger persistent increases in the firing rates of the spontaneously active medial vestibular nucleus neurons in the mouse cerebellum (Nelson et al., 2003). Strikingly, this form of neuronal plasticity requires inhibition-induced *decreases* in intracellular Ca^2+^ and CaMKII activity, which then results in reduced BK Ca^2+^-activated K^+^ channel activity that allows faster spike firing (Nelson et al., 2005). In addition, synaptic inhibition can also induce activity-dependent changes through Ca^2+^ influx triggered by post-inhibitory rebound depolarization in the postsynaptic neuron (Aizenman et al., 1998; Aizenman and Linden, 1999; Ouardouz and Sastry, 2000). Such rebound depolarization-induced Ca^2+^ influx has been shown to underlie several forms of synaptic plasticity at central inhibitory synapses (Morishita and Sastry, 1996; Aizenman et al., 1998; Pugh and Raman, 2009).

Therefore, the differences in memory formation between high and low breathing animals may be attributable to differential abilities of the conditioning stimuli to inhibit RPeD1 firing in these two groups of animals (see Figure 6). In other words, animals with higher basal respiration behavior may be predisposed to form LTM because the inhibitory inputs induced by the conditioning stimuli are able to more effectively inhibit a RPeD1 with lower firing activity, thus triggering the signaling pathways required for enhancement of firing activity and memory formation. In contrast, the same inhibitory inputs may not be able to sufficiently “pause” a RPeD1 with higher firing activity, such that their effects on the neuron may be negligible. This is consistent with our observation that despite receiving comparable numbers of conditioning stimuli in T1, only high breather animals exhibited significant reductions in the number of attempted pneumostome openings in T2 (Figure 4A). Furthermore, only LTM animals exhibited enhanced RPeD1 firing activity during the first 24 h after conditioning. Our observations support the notion that high breather animals are more likely to form LTM because the T1 conditioning stimuli were better able to trigger in the RPeD1 the requisite signaling pathways for neuronal plasticity and LTM formation.

### Memory-Specific Biphasic Enhancement in RPeD1 Firing Activity During Aversive LTM Formation

Learning-dependent enhancements in neuronal activity have been reported in the conditioned taste aversion paradigm in *L. stagnalis* (Jones et al., 2003; Kemenes et al., 2006; Nikitin et al., 2008). We observed that, during the first 24 h after conditioning, RPeD1 firing activity is enhanced in a biphasic manner only in conditioned animals that will form aversive LTM. Our findings expand upon those in a previous study using semi-intact preparation of the central ring ganglia that RPeD1 firing activity is differentially modulated within the first 3 h as compared to at 24 h after aversive operant conditioning (Braun and Lukowiak, 2011). The early enhancement of RPeD1 firing activity is likely a mechanism required for the induction of CREB1 activity in this learning paradigm (Guo et al., 2010).

Memory formation is a dynamic process, and biphasic activation appears to be a common theme in cellular and molecular processes underlying memory formation. Our findings mirror that of a previous study in one-trial classical conditioning of phototaxic behavior in the mollusk *Hermissenda*, which found that the firing activity of photoreceptor cells in conditioned animals is enhanced in a biphasic manner during the first 24 h after conditioning (Crow and Siddiqi, 1997). A number of studies have also reported two-wave activation of several memory-related proteins during memory formation, including CREB in the hippocampus after foreground contextual fear learning (Trifilieff et al., 2006, 2007), mTOR in the gustatory cortex (Belelovsky et al., 2009) and ERK/MAPK cascade in the insular cortex (Swank and Sweatt, 2001) after novel taste learning. Learning-induced enhancements in neuronal activity may serve to induce and maintain persistent activation of the activity-dependent molecules required for long-term synaptic plasticity and memory (Flavell and Greenberg, 2008). Our study characterized the time course of changes in the firing activity of a pacemaker neuron during LTM formation in an aversive learning paradigm, providing a basis for future studies into the underlying molecular mechanisms.

### Conclusion

In conclusion, our findings add to an emerging body of evidence demonstrating that the rules of plasticity in spontaneously active neurons may differ significantly from those in quiescent neurons. We have also established the aversive operant conditioning of aerial respiration in *L. stagnalis* as a well-characterized model for examining activity-dependent plasticity in spontaneously active neurons. In future studies, it would be of interest to understand whether and how manipulating spontaneous neuronal activity affects neural plasticity and memory formation. The findings may not only expand our understanding of the fundamental mechanisms of memory formation, but also suggest strategies to enhance learning in daily life and disease states.

## Author Contributions

ND and Z-PF designed the experiments; ND carried out all the experiments and analysis; ND and Z-PF interpreted the data and wrote the manuscript; Z-PF conceptualized and designed the study.

## Funding

The work was supported by an operating grant to Z-PF from the National Sciences and Engineering Research Council of Canada (NSERC: RGPIN-2014-06471). ND is a recipient of Graduate Studentship of Natural Sciences and Engineering Research Council of Canada (NSERC-CGS-M). The funders had no role in study design, data collection and analysis, decision to publish or preparation of the manuscript.

## Conflict of Interest Statement

The authors declare that the research was conducted in the absence of any commercial or financial relationships that could be construed as a potential conflict of interest.

## Abbreviations

CaMKII: Ca^2+^/calmodulin-dependent protein kinase II
CPG: Central pattern generator
CREB: cAMP response element-binding protein
ERK: Extracellular signal–regulated kinase
h: Hours
IP3i: Input 3 Interneuron
LTM: Long-term memory
LTP: Long-term potentiation
MAPK: Mitogen-activated protein kinase
min: Minute
PKA: Protein kinase A
PKC: Protein kinase C
RPeD1: Right Pedal Dorsal 1
s: Second
VD4: Visceral Dorsal 4.
